# Use of podcast technology to facilitate education, communication and dissemination in palliative care: the development of the AmiPal podcast

**DOI:** 10.1136/bmjspcare-2016-001140

**Published:** 2016-08-31

**Authors:** Amara Callistus Nwosu, Daniel Monnery, Victoria Louise Reid, Laura Chapman

**Affiliations:** 1 Marie Curie Palliative Care Institute Liverpool (MCPCIL), University of Liverpool, Liverpool, UK; 2 University Hospital Aintree, Liverpool, Merseyside, UK; 3 Royal Liverpool and Broadgreen University Hospitals NHS Trust, Liverpool, UK

**Keywords:** Technology, palliative care, audio, podcast, Education and training

## Abstract

**Objectives:**

Podcasts have the potential to facilitate communication about palliative care with researchers, policymakers and the public. Some podcasts about palliative care are available; however, this is not reflected in the academic literature. Further study is needed to evaluate the utility of podcasts to facilitate knowledge-transfer about subjects related to palliative care. The aims of this paper are to (1) describe the development of a palliative care podcast according to international recommendations for podcast quality and (2) conduct an analysis of podcast listenership over a 14-month period.

**Methods:**

The podcast was designed according to internationally agreed quality indicators for medical education podcasts. The podcast was published on SoundCloud and was promoted via social media. Data were analysed for frequency of plays and geographical location between January 2015 and February 2016.

**Results:**

20 podcasts were developed which were listened to 3036 times (an average of 217 monthly plays). The Rich Site Summary feed was the most popular way to access the podcast (n=1937; 64%). The mean duration of each podcast was 10 min (range 3–21 min). The podcast was listened to in 68 different countries and was most popular in English-speaking areas, of which the USA (n=1372, 45.2%), UK (n=661, 21.8%) and Canada (n=221, 7.3%) were most common.

**Conclusions:**

A palliative care podcast is a method to facilitate palliative care discussion with global audience. Podcasts offer the potential to develop educational content and promote research dissemination. Future work should focus on content development, quality metrics and impact analysis, as this form of digital communication is likely to increase and engage wider society.

## Background

Technology is increasingly being integrated into medicine to support new opportunities for the delivery of clinical practice, education and research.^1^ Podcasts are episodic digital audio recordings that are downloaded through web syndication or streamed online.^2^ Research demonstrates that podcast listenership is increasing.^3–5^ The percentage of Americans who have listened to a podcast has increased from 9% to 17% between 2008 and 2015.^6^ Podcasts are increasingly being used to support medical education.^7–10^ Palliative care podcasts are available;^11^ these include ‘Get Palliative Care’ (by the Center to Advance Palliative Care—CAPC),^12^ the ‘CAPC Palliative Care Podcast’^13^ and the ‘Hospice of the Bluegrass Podcast’.^14^ However, there are no published studies about the use of podcasts in palliative care. Podcasts can potentially be used to facilitate communication about palliative care with researchers, policymakers and the public.^1^ Further study is needed to evaluate the utility of podcasts to facilitate knowledge-transfer about subjects related to palliative care.

The aims of this article are to:

1. Describe the development of a palliative care podcast according to international recommendations for podcast quality.
2. To analyse the listenership of the podcast over a 14-month period.

## Methods

The development of the podcast involved defining the scope and focus of the podcast; developing an infrastructure; identifying quality indicators of podcast quality; designing content; coordinating dissemination and analysing data.

### Scope and focus

The podcast was aimed at healthcare professionals with an interest in palliative care, technology and innovation. The podcast method was chosen for its effectiveness, popularity and accessibility.^7^

### Infrastructure development

A portable audio recorder and microphone (total cost=£50) was purchased with funds from an educational grant. SoundCloud, a popular audio streaming website, was chosen to host the podcast (https://soundcloud.com/mypal). The website was accessible online and also has native applications for mobile devices (Android and iOS). An online blog was developed for the podcast (http://amaranwosu.com/amipal/) to facilitate dissemination and provide links to references presented in the podcast.

### Quality indicators

Quality indicators for medical education podcasts and blogs have been developed.^15^ These indicators were developed using a modified Delphi consensus of international healthcare professional educators. The indicators with ≥90% consensus (table 1) consist of 13 items (10 of which are relevant to podcasts) within themes that include: content, credibility, bias, transparency, academic rigour, functionality, use of resources, orientation and professionalism. These quality indicators were used to inform the podcast development.

**Table 1:** Quality indicators for medical education podcasts and blogs as recommended by Lin *et al*^15^

			Per cent consensus	
Quality indicator	Domain/subtheme	How this was met	Podcasts	Blogs
Do the authorities (eg, author, editor, publisher) that created the resource list their conflicts of interest?	Credibility/bias	There was no conflict of interest.	100	100
Is the information presented in the resource accurate?	Credibility/academic rigour	References were provided for the podcast content.	100	94
Is the identity of the resource's author clear?	Credibility/transparency	The blog and podcast included details of the affiliation and qualifications of ACN.	95	95
Does the resource make a clear distinction between fact and opinion?	Credibility/bias	The podcast and blog provided details of what constituted fact and opinion. References were provided for the podcast content.	95	95
Does the resource employ technologies that are universally available to allow learners with standard equipment and software access?	Design/functionality	The podcast was accessible using standard technologies (computer and mobiles devices) without the requirement of additional software or payment.	94	–
Does the resource clearly differentiate between advertisement and content?	Credibility/bias	The podcast was freely available and was produced without commercial funding or advertising.	90	95
Is the resource transparent about who was involved in its creation?	Credibility/transparency	Podcast production was performed by ACN. Contributions of others were clearly acknowledged.	90	91
Is the content of this educational resource of good quality?	Content	The podcasts were edited to enhance audio quality.	90	91
Is the content of the resource professional?	Content/professionalism	Each episode was planned and researched in advance to ensure the content was accurate and professional.	90	91
Is the resource useful and relevant for its intended audience?	Content/orientation	The podcast format consisted of interviews, opinion pieces and education-focused activity. The podcast was aimed at palliative care professionals who were familiar with social media.	90	91
Does the resource cite its references?	Credibility/use of other resources	References were provided for the podcast content.	–	93
Are the resources consistent with its references?	Credibility/use of other resources	References were provided for the podcast content.	–	93
Is the author well qualified to provide information on the topic?	Credibility/transparency	The blog and podcast included details of the affiliation and qualifications of ACN.	–	91

### Content design

The podcast was named AmiPal (previously MyPal), reflecting the name of the corresponding author and subject of Palliative Care. The format involved interviews, opinion pieces and education-focused content. The topics covered are presented in table 2. Podcasts were edited using Audacity (http://www.audacityteam.org), a free open-source, cross-platform audio-editing tool.

**Table 2:** Topics covered in AmiPal podcasts since January 2015

Topic	Focus	Length	Date published
Introduction and welcome to the new podcast	Opinion	12:02	Jan 2015
Research and innovation	Opinion	17:22	Jan 2015
Integrated clinical academic training	Article overview	6:13	Jan 2015
Nanotechnology to monitor cancer	Opinion	9:34	Jan 2015
3D printing in clinical practice	Opinion	7:15	Jan 2015
Publishing in palliative care	Education	15:19	Feb 2015
Is there too much technology in healthcare	Article overview	14:55	Feb 2015
Peer led learning in palliative care	Article overview	5:35	Mar 2015
Palliative care day therapy	Interview	21:42	Mar 2015
Undergraduate medical education in palliative care	Interview	15:31	Mar 2015
Bioelectrical impedance analysis to assess hydration in advanced cancer	Education	6:14	Mar 2015
Culture and palliative care	Opinion	16:27	May 2015
Wearable technology in healthcare—can palliative care benefit?	Opinion	14:10	Jun 2015
Five apps for clinical academics	Education	16:40	Jun 2015
Social media and palliative care	Article overview	4:10	Sep 2015
Technology in the delivery of healthcare: patient power in medicine	Article overview	3:44	Nov 2015
What makes a good case-based discussion?	Interview Education	5:37	Dec 2015
Virtual reality and palliative care	Opinion	5:48	Feb 2016
Renal medicine and palliative care	Interview	3:36	Feb 2016
A comparison between studies: research, audit and service evaluation	Education	2:22	Feb 2016

### Dissemination

The podcasts were released episodically under the ‘Science and Medicine’ category on the SoundCloud website. The podcast's Rich Site Summary (RSS) feed was registered with podcast repositories, including iTunes (http://www.apple.com/itunes), Stitcher (https://www.stitcher.com), TuneIn (http://tunein.com) and Acast (https://www.acast.com). The RSS feed enabled users to access the podcast via a computer or mobile device. Each episode was promoted on social media using palliative medicine hashtags.^16^ Widgets (stand-alone embeddable web applications) were embedded into the blog and social media posts, which enabled the podcasts to be directly played.

### Analysis and feedback

Feedback to each episode was possible using email communication and social media. Additionally, healthcare professionals (in Merseyside, UK) were contacted by email and were encouraged to provide feedback. The listenership analysis was conducted using the SoundCloud analytics tools. Data were analysed for frequency of plays and geographical location.

## Results

**Figure 1:**
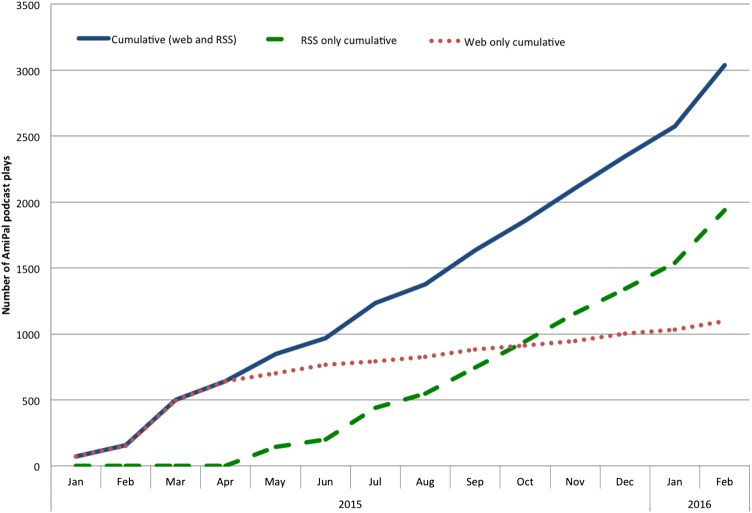
Line chart displaying the total number of times the AmiPal podcast was listened to between January 2015 and February 2016 via the SoundCloud web and Rich Site Summary-feed options.

Twenty podcasts were developed between January 2015 and February 2016. The cumulative total of podcast plays was 3036, an average of 217 monthly plays (table 3 and figure 1). The RSS feed was the most popular way to access the podcast (n=1937; 64%). Between January and September 2015, the podcast was most accessed via the SoundCloud website. However, from October 2015, the cumulative RSS feed plays were higher. The mean duration of each podcast was 10 min (range 3–21 min). The podcast was listened to in 68 different countries (table 4) and was most popular in English-speaking areas; specifically, the USA (n=1372, 45.2%), UK (n=661, 21.8%) and Canada (n=221, 7.3%).

**Table 3:** Number or times the AmiPal podcast was played, via the web and RSS feed options, between January 2015 and February 2016

			Number of times AmiPal podcast was played (n)				
Year	Month	Web only	Web only cumulative	RSS only	RSS only cumulative	Monthly total (web + RSS)	Total cumulative
2015	Jan	71	71	0	0	71	71
	Feb	84	155	0	0	84	155
	Mar	344	499	0	0	344	499
	Apr	144	643	0	0	144	643
	May	61	704	143	143	204	847
	Jun	66	770	55	198	121	968
	Jul	25	795	241	439	266	1234
	Aug	34	829	107	546	141	1375
	Sep	56	885	201	747	257	1632
	Oct	30	915	195	942	225	1857
	Nov	34	949	217	1159	251	2108
	Dec	56	1005	183	1342	239	2347
2016	Jan	29	1034	197	1539	226	2573
	Feb	65	1099	398	1937	463	3036

**Table 4:** Top 10 geographical locations for AmiPal podcast listeners

Position	Country	Number of podcast plays (%)
1	USA	1372 (45.2)
2	UK	661 (21.8)
3	Canada	221 (7.3)
4	Australia	217 (7.1)
5	Brazil	164 (5.4)
6	New Zealand	69 (2.3)
7	Germany	38 (1.3)
8	India	26 (0.9)
–	The Netherlands	26 (0.9)
9	Ireland	20 (0.7)
10	Malaysia	17 (0.6)
–	Fifty-seven other countries	205 (6.8)

A small amount of feedback was received (10 responses); overall, this was positive. The podcast was modified in response to the feedback with changes to the audio quality, style and format. Specifically, the podcast length shortened to <6 min (evident from the last six podcasts) and backing music was added to improve the rhythmic flow of the audio.

## Discussion

### Summary

This analysis demonstrated that the AmiPal palliative care podcast had a wide geographical reach with the majority of listeners originating from Western English-speaking countries.

### Strengths and uniqueness of this study

This is the first study that describes the development and analysis of a palliative care podcast that was developed according to relevant quality indicators. The podcast was free and accessible across a range of computer and mobile platforms.^9^ The data of the geographical reach of the podcast provide evidence of the potential of this medium to facilitate international dissemination.

### Comparison with previous work

Previous studies have highlighted potential to use technology to inform education and dissemination in palliative care.^1^ This study adds to evidence from other work, which have used podcasts in medical education.^8^ ^10^ ^17^ The podcasts were accessed and played several months after release, which may suggest that new listeners were acquired over-time, and/or the archive was used ‘on-demand’. These findings are consistent with previous work, which reports how podcasts provide a repository of information that can be continually accessed.^2^ ^18^ The majority of podcasts (64%) were accessed via the RSS feed, which may suggest the use of mobile devices. This finding is consistent with the findings of USA and UK research, which demonstrates that two-thirds of podcasts are accessed on a mobile device rather than a computer.^4^ ^19^ The podcast listenership was similar to the CAPC podcast, which (at the time of writing) has a total of 3831 listens from its 12 episodes over the past 24 months. In 2015, CAPC's public facing ‘Get Palliative Care’ podcast series obtained 14 318 listens from 10 podcasts about the patient journey. This highlights the potential interest for podcasts reporting the patient narrative.

### Limitations

The lack of plays from the RSS feed in the first 4 months was due to a delay in the RSS feed being available. Consequently, the potential reach of the podcast in these months was lower. It is likely that the overall proportion of RSS feed plays would have been higher, if the RSS feed was available for the entire period. It is likely that the majority of the RSS feed plays were from mobile devices; however, we cannot ascertain the exact number (as the RSS feed may have been accessed by computer). Furthermore, it is not possible to know whether users listened to the entire podcast or not. Although the podcast was available across a range of computer and mobile devices, there may be some technological challenges to accessing the podcast in some healthcare organisations and resource-poor settings (eg, old internet browsers, web-filtering issues, wireless internet coverage).

Very little feedback was received through the email and social media feedback options. A possible explanation, presented by experts in medical education, may be that the listeners did not place importance on interacting with the podcast host.^15^ Listeners may personally reflect on the podcast topics without feeling the need to communicate their reflections with the host. Consequently, it is not possible to determine if listeners found the podcasts beneficial. Furthermore, our knowledge of the listenership is relatively unknown, as listeners were not required to provide information or login to access content.

### Implications to practice

It is possible to develop a palliative care podcast that has a global reach. Audio recording equipment is available for relatively low cost,^20^ and many mobile devices contain microphones to record audio.^21^ Audio hosting sites (eg, SoundCloud.com, Podomatic.com) and open-source audio editing software are freely available (eg, Audacity).^20^ ^21^ Individuals and organisations planning on developing their own podcasts can use quality indicators^15^ ^22^ to develop content and social media to enhance dissemination.^16^ ^20^ If wide dissemination of the podcast is intended, the RSS feed should be registered with podcast databases and social media should be used for promotion.

### Future opportunities and research possibilities

Organisations may consider developing podcasts for specific purposes, such as education, lecture capture and research dissemination. Future studies are needed to determine whether palliative care podcasts can facilitate learning for professionals and lay people. Further work can examine the demographics of listeners (eg, using analytics software and surveys) and evaluate learning outcomes of podcasts using of pre and post assessments; this will help to plan priorities for content, quality and to evaluate the impact (eg, for learning and clinical practice) of podcasts. Developed content can be incorporated within the dissemination strategy of institutions, in order to meet learning styles of listeners. Future work can also consider the needs of individuals with hearing deficits (eg, via subtitle video).

## Conclusions

Podcasts can be used to facilitate palliative care discussion with a global audience. Podcasts offer the potential to develop educational content and promote research dissemination. Future studies should focus on information development, quality metrics and impact analysis of educational podcasts, as this form of digital communication is likely to increase and engage wider society.

## Ethics

This project did not constitute research. Therefore, ethics committee approval was not required.
